# Emotional dynamics and hearing differentiation under a unified procedure: a field study of grassroots procuratorial hearings in China

**DOI:** 10.3389/fpsyg.2026.1912074

**Published:** 2026-08-26

**Authors:** Dong Liang, Xiaoming Tian, Siyi Sun

**Affiliations:** 1 School of Language, Literature and Law, Xi'an University of Architecture and Technology, Xi'an, China; 2 School of Humanities and Foreign Languages, Xi'an University of Posts and Telecommunications, Xi'an, China

**Keywords:** China, emotional dynamics, grassroots procuratorial hearings, hearing differentiation, judicial ritual

## Abstract

**Introduction:**

Grassroots procuratorial hearings in China operate under nationally standardized procedural regulations intended to ensure consistency and procedural uniformity. Yet hearings conducted within the same institutional framework often unfold in different ways. Existing studies have primarily explained procuratorial hearings through institutional arrangements, procedural functions, and ritual authority, providing limited insight into the micro-level processes through which such variation emerges. This study examines how emotional dynamics shape the differentiation of grassroots procuratorial hearing practices.

**Methods:**

The study draws on immersive qualitative fieldwork involving 20 grassroots procuratorial hearings conducted across eight district-level procuratorates in a provincial capital city. Data include field notes, hearing transcripts, observational records, and anonymized case materials. Guided by a four-dimensional analytical framework consisting of emotional arousal, emotional expression, emotional negotiation, and affective empathy, the study employs abductive thematic analysis to identify recurring patterns of interaction.

**Results:**

The findings suggest that standardized procedures provide merely the institutional framework within which hearings unfold. Different forms of hearing practice emerge through distinct configurations of emotional processes. Three relatively stable interactional patterns were identified: assembly-line compliance, gladiatorial confrontation, and negotiated empathy. Among the four dimensions, emotional negotiation emerged as the key mechanism linking individual emotional experiences to collective interactional outcomes. The framework also explains variations in hearing officers’ participation, ranging from procedural detachment to active empathic engagement, as well as atypical cases that do not fully conform to dominant patterns.

**Discussion:**

This study develops an emotion-centered perspective for understanding grassroots procuratorial hearings and offers a unified explanation for hearing differentiation under a common procedural framework. Foregrounding emotional dynamics as a central component of judicial interaction, it contributes to research on judicial rituals and judicial emotions while providing practical insights for improving grassroots hearing practices.

## Introduction

Grassroots procuratorial hearings constitute an institutionalized procedural arrangement within China’s judicial system. To standardize hearing practices nationwide, top judicial authorities have issued unified hearing regulations and standardized procedures that specify the scope of applicable cases, the composition of participants, procedural stages, and deliberation rules. From the perspective of institutional design, grassroots hearings across different regions are expected to follow a common procedural script and exhibit relatively uniform operational patterns and interactional rhythms.

However, field observations reveal a striking discrepancy between institutional expectations and practical realities. The uniformity embodied in institutional texts has not translated into homogeneous practices on the ground. Within an identical legal and procedural framework, grassroots hearings display substantial variation in interactional processes and outcomes. Some hearings unfold as routine procedural exercises with limited participant engagement, while others become emotionally charged and highly contested. In still other cases, participants engage in extensive dialogue, mutual understanding, and constructive communication. Procuratorial hearing practices in China exhibit substantial selective application and regional variation, as demonstrated by a nationwide analysis of 41,896 hearing decisions on non-prosecution cases (Lu, 2023). However, quantitative evidence alone cannot explain why distinct forms of hearing practice emerge under the same procedural framework, particularly from the perspective of micro-level interactional processes. The emergence of different hearing trajectories under a common institutional script therefore constitutes an empirical puzzle that warrants further investigation.

Before examining these three institutional features, it is necessary to clarify the meaning of Chinese procuratorial hearings. Procuratorial hearings refer to a procedural mechanism through which procuratorial organs, during the handling of cases, convene hearing officers, parties, and other relevant participants to openly solicit opinions on issues including fact determination, legal application, and case disposition, and use these opinions as a basis for making or assisting in judicial decisions. Unlike jury trials or judge-led proceedings in Western legal systems, procuratorial hearings in China are neither adjudicative procedures dominated by lay participation nor merely fact-finding meetings. Rather, they represent an institutional arrangement through which procuratorial organs introduce external participation and incorporate social opinions into the process of case review.

To facilitate international readers’ understanding of the research setting, it is necessary to clarify three institutional features that distinguish Chinese procuratorial hearings from judicial practices in Western legal systems. First, procuratorial hearings in China serve as an important mechanism through which procuratorial organs review cases and strengthen legal supervision, combining both judicial and social governance functions [Supreme People’s Procuratorate of the People’s Republic of China (SPP), 2020]. Second, hearing officers are neither jurors nor randomly selected citizens but community elites, professionals, and retired practitioners selected and certified by judicial authorities, thereby forming a relatively stable tripartite interactional structure involving prosecutors, parties, and hearing officers (Tan and Chen, 2022). In addition to hearing officers, participants typically include case-handling prosecutors, criminal suspects or defendants, victims and their legal representatives, defense counsels, litigation representatives, and representatives from relevant administrative agencies or enterprises. Where necessary, experts, appraisers, or guardians of juvenile participants may also participate. Third, procuratorial hearings are designed not only to facilitate legal decision-making and procedural legitimacy but also to resolve disputes, alleviate emotional tensions, and repair damaged social relationships. Procuratorial hearings provide a platform for equal dialogue among multiple parties, contributing to conflict resolution, improving litigation efficiency, and expanding opportunities for public participation in procuratorial work (Bai et al., 2024).

Procuratorial hearings may be initiated either proactively by procuratorial organs based on their authority or upon applications submitted by parties, defense counsels, litigation representatives, or relevant organizations. Their application scope covers a wide range of matters, including cases involving decisions on non-prosecution, reviews of the necessity of detention, criminal petitions, civil and administrative supervision, public interest litigation, community corrections, parole, and temporary execution outside prison. In terms of outcomes, hearings themselves generally do not directly produce final decisions; rather, they generate hearing opinions that serve as references for procuratorial decision-making. Common outcomes include decisions of relative non-prosecution or conditional non-prosecution, initiation of prosecution accompanied by sentencing recommendations, maintenance or correction of previous decisions, issuance of procuratorial recommendations to administrative agencies, approval or rejection of specific applications submitted by parties, and facilitation of mediation or settlement agreements between relevant parties. Therefore, procuratorial hearings perform multiple functions in practice, including case review, dispute resolution, social supervision, and legitimacy justification.

Existing scholarship on procuratorial hearings in China has primarily examined the institution from the perspectives of institutional arrangements, procedural functions, and ritual authority, with particular attention to the legal and symbolic foundations of the hearing system (e.g., Bai et al., 2024; Tan and Chen, 2022). Relatively little attention has been paid to the concrete interpersonal interactions that unfold during hearings, especially the emotional processes through which participants interpret situations, respond to one another, and shape the course of interaction. As a result, existing research lacks a coherent analytical framework capable of linking emotional dynamics to broader patterns of hearing practice. Consequently, it remains difficult to explain a central empirical puzzle: Why do different forms of hearing practice emerge under the same legal and procedural framework? To address this gap, this study shifts attention to the micro-level dynamics of hearing interaction and examines how different configurations of emotional processes shape the differentiation of grassroots procuratorial hearing practices.

This study moves beyond conventional analyses centered on institutional structures and procedural rules to examine the interpersonal processes through which hearing practices are produced in everyday interaction. Drawing on immersive qualitative fieldwork, it develops an analytical framework focusing on emotional arousal, emotional expression, emotional negotiation, and affective empathy, and explores how different configurations of these emotional processes generate divergent forms of hearing practice under a unified institutional framework. Particular attention is paid to the mechanisms through which hearing trajectories emerge, evolve, and stabilize in practice. Accordingly, the study addresses three research questions:

*RQ1:* How do distinct forms of hearing interaction emerge despite a unified institutional and procedural framework?

*RQ2:* How do emotional interactions shape participants’ behavior, interpersonal relationships, and the overall trajectory of hearings?

*RQ3:* How do emotional dynamics help explain atypical hearing trajectories and variations in the participation of hearing officers?

## Literature review

Existing studies of procuratorial hearings have primarily developed along two research traditions. The first is the doctrinal legal approach, which focuses on institutional design, procedural regulations, supporting mechanisms, and practical implementation (Huo, 2022). Research within this tradition seeks to improve hearing practices through procedural refinement, process optimization, and enhanced supervision. Similar concerns can also be found in international discussions of citizen participation in prosecution review systems. For example, studies of Japan’s Prosecution Review Commission have examined how citizen involvement functions as a mechanism for supervising prosecutorial power, while emphasizing the operation of formal institutional arrangements (Johnson and Hirayama, 2019). Although this body of research provides important insights into the design and regulation of hearing systems, it pays relatively little attention to what occurs during hearings themselves, particularly the interactional processes through which participants interpret, negotiate, and enact institutional rules.

The second tradition is the socio-legal approach. Moving beyond a purely doctrinal perspective, socio-legal scholars have introduced sociological concepts to examine judicial institutions and legal practices. Among these approaches, Bourdieu’s (1977) concepts of *field, habitus, and capital* have been widely used to analyze the production of judicial authority and the reproduction of institutional order (Madsen and Dezalay, 2002). Other studies have interpreted judicial proceedings through the lenses of ritual performance, symbolic power, and cultural narratives (Li, 2021; Van Oorschot, 2021). These perspectives have expanded our understanding of the social foundations of legal institutions. However, their primary analytical focus remains on institutional structures, symbolic meanings, and broader patterns of social order. As a result, the concrete interactional processes through which participants construct relationships, respond to one another, and shape hearing dynamics often remain underexplored.

A common limitation of both traditions is their emphasis on macro-level explanations. Existing research has generated substantial knowledge about procedural design, institutional legitimacy, and symbolic authority, but has been less successful in explaining why hearing practices often diverge despite operating under the same formal rules. The persistence of variation under conditions of procedural uniformity suggests that factors beyond institutional arrangements may play an important role in shaping hearing outcomes. Understanding this variation requires closer attention to the micro-level processes through which participants interact within hearing settings.

Recent developments in socio-legal research have increasingly highlighted the importance of interaction and emotion in judicial settings. Studies of courts, mediation, and community corrections have demonstrated that legal processes are not governed solely by formal rules but are also shaped by emotion management, emotional labor, and interpersonal coordination (Leben, 2019). Research has shown that participants’ responses to legal procedures are influenced by their emotional experiences, perceptions of fairness, and relationships with other actors involved in the process (Maroney and Gross, 2014). These findings suggest that emotions are not peripheral to judicial practice but constitute an important dimension of legal interaction.

The growing attention to micro-level interaction has also generated new insights into the operation of judicial rituals. Studies of grassroots justice have found that standardized procedures can produce different practical outcomes through variations in role performance, narrative construction, and interpersonal interaction (Roach Anleu and Mack, 2017; Van Oorschot, 2021). Similarly, research on online court hearings has demonstrated that changes in interactional conditions can reshape participants’ experiences and alter the forms through which judicial authority is enacted (Collins, 2004; Rossner and Tait, 2024). These studies indicate that judicial processes cannot be fully understood through institutional design alone and that interactional dynamics deserve closer examination.

Despite these developments, research on procuratorial hearings has rarely paid attention to how the emotional exchanges among prosecutors, hearing officers, suspects, victims, and other participants shape the course of hearings. Building on the research gap, this study focuses on emotional interaction as a central mechanism shaping hearing practices. Specifically, this study examines how emotional dynamics are generated, negotiated, and transformed during hearings and how these processes influence participants’ behavior, relationships, and interactional environments. By analyzing both typical and atypical hearing practices through a unified interactional perspective, the study seeks to explain how practical differentiation emerges within a formally standardized institutional setting.

## Theoretical considerations

This study develops an analytical and explanatory framework consisting of four dimensions: emotional arousal, emotional expression, emotional negotiation, and affective empathy. Rather than integrating multiple theories into a single explanatory model, the framework draws selectively on insights from the sociology of emotions, social psychology, and interactional sociology as sensitizing concepts for interpreting emotional processes observed during fieldwork. Emotional arousal is informed by Russell’s (2003) insights of core affect, emotional expression draws on Hochschild’s (1983) work on emotion management and emotional labor, emotional negotiation is developed inductively from the empirical data while being informed by research on emotions as social information (van Kleef, 2009, 2016), and affective empathy is informed by studies of empathy and compassion (Decety and Jackson, 2004; Klimecki, 2019). These concepts are treated not as separate theories but as analytically distinct dimensions of emotional interaction operating at different stages of the hearing process. They provide a coherent framework for examining how emotions emerge, become expressed, are negotiated among participants, and ultimately shape interactional outcomes in grassroots procuratorial hearings.

Emotional arousal refers to the activation of emotional states in response to external stimuli and personal experiences. Emotional experiences can be understood through the dimensions of valence and arousal, while emotional intensity varies according to individuals’ subjective evaluations of events and situations (Russell, 2003; Zou et al., 2022). In the context of procuratorial hearings, participants rarely enter the hearing room as emotionally neutral actors. Their emotions are often shaped by prior case experiences, perceived gains and losses, family circumstances, procedural expectations, and previous interactions with judicial institutions. Emotional arousal therefore serves as a starting point for understanding the emotional baseline that participants bring into hearings and helps explain variations in emotional tension across different cases.

Emotional expression refers to the observable manifestation of emotions through verbal and nonverbal behavior. Research in the sociology of emotions suggests that emotional expression is not simply a spontaneous reflection of inner feelings but is often shaped by situational norms and emotion management processes (Hochschild, 1983; Turner, 2007). At the same time, emotions perform important communicative functions in social interaction, influencing how individuals interpret situations and respond to others (Zaki and Williams, 2013). In this study, emotional expression is used as an empirical category for analyzing participants’ behavioral responses during hearings. Based on field observations, restrained expression includes silence, lowered gaze, and minimal verbal participation; confrontational expression includes argumentation, direct challenges, and displays of anger; narrative expression involves personal storytelling and accounts of life difficulties. These forms of expression constitute the primary channels through which emotions become visible in hearing interactions.

While emotional expression concerns the outward presentation of emotions, emotional negotiation refers to the interactional processes through which participants respond to, adjust to, and strategically engage with one another’s emotional displays. This concept emerged inductively from field observations and is informed by research on emotions as social information (van Kleef, 2009, 2016). According to this perspective, emotional expressions provide information that influences how others interpret situations and make behavioral adjustments. In hearings, emotional negotiation occurs when prosecutors, hearing officers, parties, and other participants continuously assess emotional cues and modify their responses accordingly. Through questioning strategies, narrative exchanges, conciliatory gestures, or confrontational responses, participants actively shape the emotional atmosphere of the hearing. Emotional negotiation therefore captures the dynamic and relational nature of emotional interaction that cannot be reduced to individual emotional states alone.

Affective empathy refers to the emergence of emotional resonance and compassionate understanding between participants. Existing research suggests that empathy and compassion can facilitate prosocial behavior, reduce interpersonal hostility, and increase willingness to cooperate and reconcile (Decety and Jackson, 2004; Klimecki, 2019). In the context of procuratorial hearings, affective empathy occurs when participants move beyond formal role expectations and become emotionally receptive to the experiences and circumstances of others. Such processes may be observed when hearing officers acknowledge personal hardships, when prosecutors demonstrate understanding toward parties’ situations, or when participants recognize one another’s emotional concerns. The presence or absence of affective empathy plays an important role in shaping whether hearings become confrontational, procedural, or reconciliatory in character.

The four dimensions are analytically distinct but closely interconnected. Rather than operating independently, they form a sequential process through which emotional dynamics unfold during hearings. Participants enter hearings with different levels of emotional arousal that stem from their prior experiences and expectations. These emotions become observable through various forms of emotional expression. As interaction progresses, participants engage in emotional negotiation by responding to and adjusting their behavior in relation to others’ emotional displays. Depending on how these interactional processes develop, affective empathy may emerge, remain limited, or fail to occur altogether. Different configurations of these emotional mechanisms contribute to the formation of relatively stable interactional patterns and help explain variations in hearing practices under a formally unified procedural framework.

## Methodology

This study adopts a multi-sited qualitative fieldwork approach. The investigation covers eight district-level procuratorates within a provincial capital city, encompassing the routine practice settings of procuratorial hearings across the municipal jurisdiction. During the research period, a total of 20 formal hearings were observed, participated in, and documented (see Appendix A: Case Overview for details). The cases include major statutory categories such as non-prosecution review, review of coercive measures, civil and administrative supervision, and public interest litigation. Case selection followed a theoretically informed sampling strategy aimed at maximizing variation in interactional contexts. Rather than pursuing statistical representativeness, the study deliberately included hearings involving diverse dispute backgrounds, litigants’ life circumstances, and varying degrees of interpersonal tension. The sample therefore encompasses highly adversarial encounters, routine procedural hearings, and negotiation-oriented interactions, providing rich empirical materials for examining how emotional processes unfold across different hearing settings.

The first author participated in all 20 hearings in the capacity of an officially certified municipal hearing officer with cross-jurisdictional authorization. This position provided access not only to formal hearing sessions but also to pre-hearing consultations among prosecutors and post-hearing deliberations. Such immersive access enabled close observation of the micro-level dynamics of hearing interactions, including changes in tone, facial expressions, bodily posture, conversational rhythm, and situational atmosphere. Particular attention was paid to moments of tension, accommodation, hesitation, conflict, and emotional adjustment among participants. Immediately following each hearing, detailed field notes were compiled to document interactional sequences, emotional atmospheres, and the evolving relationships among participants. These materials preserved aspects of hearing practice that are often inaccessible through official records alone.

The second and third authors were primarily responsible for research design, theoretical development, and the interpretation of field materials. After each field record was completed, the two authors reviewed field notes, hearing transcripts, and observational records, identifying interaction episodes that appeared analytically significant while removing procedural redundancies. Particular attention was given to moments involving emotional tension, behavioral adjustment, shifts in interpersonal relationships, and changes in the hearing atmosphere. Through repeated comparison across cases, recurring emotional processes gradually became visible. The analytical dimensions of emotional arousal, emotional expression, emotional negotiation, and affective empathy were subsequently refined as sensitizing concepts for organizing and interpreting these observations rather than serving as predetermined coding categories.

This fieldwork was conducted based on a formally authorized hearing officer role, with all procedures completed in accordance with judicial regulations and academic ethical standards. The research process relied on a collaborative workflow integrating immersive field observation and analytical interpretation. The first author focused on documenting the unfolding interactional realities of hearings, preserving the complexity, ambiguity, and spontaneity of participants’ conduct. The second and third authors focused on analytical interpretation, comparing observations across cases, identifying recurring interactional patterns, and developing conceptual explanations grounded in the empirical materials. Through iterative dialogue, the three authors jointly reviewed field records, clarified ambiguous observations, reconstructed uncertain interaction sequences, and refined analytical interpretations, ensuring that the findings remained closely connected to the empirical realities of hearing practice.

The first author’s participation as a hearing officer involved a dual role that requires particular reflexive consideration. As a hearing officer, the first author occupied an institutionally visible position, and prosecutors or parties may have adjusted their verbal and emotional expressions in response to this presence, for example, by adopting more restrained or formal ways of speaking or by seeking recognition from the hearing officer. To minimize such effects, the first author primarily focused on observation and documentation during hearings and intervened only when required by the responsibilities of a hearing officer, such as maintaining order, clarifying factual issues, or expressing hearing opinions. All intervention-related statements were fully recorded in the field notes and treated as data requiring careful interpretation during subsequent analysis. In addition, the second and third authors did not participate in field observations but independently reviewed the empirical materials and engaged in theoretical interpretation. Their regular discussions with the first author helped identify and correct potential selective attention resulting from the first author’s institutional role. It should be acknowledged that this dual role may have generated certain observer effects; however, it also enabled access to interactional spaces that would have been difficult for external observers to enter, allowing a more nuanced understanding of the micro-level processes through which hearing interactions unfolded.

The empirical materials consist primarily of first-author field notes, informal observational records collected before and after hearings, officially archived hearing transcripts, and anonymized procedural case documents. These materials capture hearings across multiple temporal stages and observational perspectives, documenting procedural developments, interpersonal interactions, and changes in emotional atmosphere. Rather than assuming mutual verification among different sources, the study treats each form of data as offering a distinct perspective on the interactional dynamics of hearings.

Data were analyzed using an abductive thematic analysis approach. Following the analytical logic outlined by Thompson (2022), the analysis proceeded through an iterative movement between empirical observation and theoretical interpretation. The analytical process consisted of three stages. In the first stage, the three authors independently reviewed field notes, hearing transcripts, and observational records and identified interactional events related to emotional processes. In the second stage, recurring interactional patterns were identified through repeated cross-case comparison. In the third stage, these patterns were interpreted through the four dimensions of emotional arousal, emotional expression, emotional negotiation, and affective empathy to explain their underlying mechanisms. Throughout this process, the four-dimensional framework functioned as sensitizing concepts rather than predetermined coding categories.

Initial coding remained closely grounded in field notes, transcripts, and observational records, focusing on interactional events, emotional responses, behavioral adjustments, and changes in participant relationships. Through repeated comparison across hearings, recurring patterns of emotional dynamics gradually emerged. These patterns were subsequently interpreted through the sensitizing concepts of emotional arousal, emotional expression, emotional negotiation, and affective empathy. This approach avoids imposing rigid theoretical categories on empirical materials while maintaining a productive dialogue between conceptual sensitivity and field-based discovery.

The identification of the four analytical dimensions was based on operational definitions developed from observable interactional features in hearing practices. Rather than treating emotional processes as abstract psychological states, this study examined how they became recognizable through participants’ verbal expressions, interactional adjustments, and shifts in conversational patterns. The operationalization of these emotional processes and their corresponding observable indicators are summarized in Table 1.

**Table 1:** Operationalization of emotional processes in procuratorial hearing interactions.

Dimension	Core focus	Observable interactional indicators
Emotional arousal	Participants’ initial emotional states	Affective vocabulary, references to family or economic pressures, changes in tone
Emotional expression	External manifestations of emotional states	Response length, vocal intensity, interruptions, silence, and narrative styles
Emotional negotiation	Interactive adjustment of emotional responses	Changes in questioning strategies, response patterns, and interactional rhythms among prosecutors, parties, and hearing officers
Affective empathy	Emergence of emotional understanding and relational connection	Shifts from procedural questioning to supportive inquiry and from factual statements to personal narratives

Beyond identifying interactional events and emotional atmospheres, the analysis also closely examined participants’ discursive practices, including questioning structures, response length, interruptions and overlaps, positions of silence, degrees of narrative elaboration, shifts in tone, and evaluative expressions. These linguistic features were treated as observable manifestations of emotional arousal, emotional expression, and emotional negotiation, providing empirical evidence for distinguishing among the three interactional patterns.

For example, in Case 8, the participant’s statement, *“Both of my children are still young. The older one is twelve, and the younger one is in the final year of kindergarten. I have to support both of them,”* was identified as an expression of emotional arousal and family responsibility narratives. The prosecutor’s response, *“I understand your situation, but objectively speaking, you did violate the supervision requirements,”* demonstrated an accommodative adjustment in emotional negotiation. The subsequent inquiry by the hearing officer into the participant’s family circumstances reflected the involvement of affective empathy. A single interactional event may involve multiple dimensions simultaneously; therefore, pattern identification relied on the overall similarity of dimensional configurations across multiple interactional episodes.

The entire process of fieldwork and data analysis adhered to established ethical standards for social science research. All regional identifiers, institutional markers, personal information, and case-specific details were fully anonymized. Original field notes, transcripts, and supporting materials are securely archived and accessible only to the research team for academic purposes. These measures ensure confidentiality and prevent identifiable disclosure while preserving the integrity and authenticity of the empirical record.

The findings were developed through repeated comparison, discussion, and joint interpretation among the authors. Rather than classifying cases according to predetermined theoretical categories, the analysis began with observed similarities and differences across hearing interactions. Through iterative examination of the empirical materials, several recurring patterns of emotional dynamics became apparent. The concepts of emotional arousal, emotional expression, emotional negotiation, and affective empathy were then theorized and employed as analytical lenses for interpreting these patterns and explaining how different forms of hearing interaction emerged within a formally standardized procedural framework. This analytical strategy ensured close alignment between empirical observation, conceptual interpretation, and theoretical explanation throughout the study. To enhance the transparency and robustness of the analytical process, the three authors regularly discussed disagreements in coding and interpretation and conducted focused analyses of deviant cases that did not fully correspond to the dominant interactional patterns.

## Findings

Across the 20 grassroots procuratorial hearings conducted across eight district-level procuratorates, three relatively stable forms of hearing interaction became apparent. Although all hearings followed the same procedural requirements, they differed in emotional atmosphere, interactional rhythm, participant involvement, and the extent to which mutual understanding developed among those present.

### The assembly-line compliance pattern

The assembly-line compliance pattern is characterized by low levels of emotional arousal, restrained forms of emotional expression, minimal emotional negotiation, and little evidence of affective empathy. Hearings displaying this pattern commonly involved juvenile offenses, minor criminal cases, or routine supervisory matters in which factual disputes were limited and anticipated outcomes were relatively predictable. Participants generally entered the hearing with modest emotional investment in the proceedings. Emotional tension remained low throughout the interaction, and the hearing unfolded primarily as a procedural exercise rather than a site of active interpersonal engagement.

A notable feature of this pattern is the limited visibility of emotion in interaction. Participants typically remained quiet, avoided prolonged eye contact, and spoke only when invited to do so. Their shoulders were often slightly hunched, their gaze directed toward the table or floor, and their responses brief and subdued. When questioned, they frequently paused before speaking, offering concise answers without elaboration. Even after prosecutors announced proposed decisions or evaluative comments, visible emotional reactions were uncommon. Slight nods, short acknowledgements, and prolonged silences often replaced more expressive forms of response. Emotional expression, when present, remained highly restrained.

The low level of emotional expression was accompanied by an absence of substantive emotional negotiation. Participants rarely attempted to test the reactions of prosecutors or hearing officers, nor did they actively seek emotional recognition or support. Silence functioned less as resistance than as acquiescence. Prosecutors, for their part, maintained a steady procedural rhythm. Questions followed a standardized sequence and were delivered in a measured tone. Little effort was made to explore emotional concerns or adjust questioning strategies in response to participants’ emotional cues. Once an answer was received, the hearing moved directly to the next procedural step. Interaction remained courteous but distant, producing a hearing atmosphere marked by emotional neutrality and procedural efficiency.

These interactional features were reflected in participants’ verbal practices. For example, in Case 1, when the hearing officer asked about the juvenile participants’ educational and family backgrounds, one participant briefly responded: *“I did fairly well in primary school, but gradually fell behind after entering middle school…”* The participant continued, *“Every time I called her, she would simply tell me to eat well, dress warmly, and study hard. It was always the same few words, and after a while, I became reluctant to call her.”* The prosecutor did not further explore the emotional content embedded in this statement and instead moved directly to the next procedural step. In Case 4, when asked whether they understood that their conduct violated the law, the three juveniles, respectively, provided brief responses: *“I knew it was definitely wrong, but we only took some cigarettes and beverages, and the value was not significant”*; *“I knew it was an illegal act. At that time, I just wanted to get some money to spend and did not think about anything else.”* In Case 6, when asked whether he understood that lending his bank card to others could potentially constitute a crime, the participant responded in a quiet and fearful voice: *“Um… I knew it was definitely unlawful, but I did not know it would be considered a crime.”* He then quietly asked: *“Will I be expelled from school? Will I go to prison? Then everything will be over.”* These brief, fact-oriented, and emotionally restrained responses were coded as restrained emotional expression, while prosecutors’ practice of not pursuing the emotional content of participants’ statements and moving directly to subsequent procedural steps was coded as minimal emotional negotiation. Together, these two features constituted the core configuration of the assembly-line compliance pattern.

Hearing officers displayed a similarly restrained form of participation. Most remained focused on documentary materials, occasionally taking notes while paying limited attention to participants’ emotional expressions. Eye contact was infrequent, communication among hearing officers was minimal, and interventions were rare. Their role was largely confined to observing the formal progress of the hearing rather than shaping its interpersonal dynamics. Consequently, opportunities for emotional negotiation or affective empathy remained limited.

Cases 1, 4, 6, and 15 most clearly illustrate this interactional pattern. These hearings involved matters such as juvenile group fighting, petty theft, cyber-related offenses, and routine public-safety supervision. During one juvenile hearing, the young participant sat with his head lowered throughout the proceedings, repeatedly intertwining his fingers in his lap. When his guardian spoke on his behalf, he remained silent and avoided looking toward the prosecutor. Even after receiving a formal admonition, he merely nodded slightly without offering any verbal response. Similar forms of restraint appeared across the other cases. Participants generally limited themselves to answering questions, rarely volunteered personal narratives, and showed little interest in contesting procedural conclusions. The interaction moved forward smoothly but generated little interpersonal engagement beyond what was required by the hearing procedure itself.

Although this pattern was generally stable, occasional variations revealed subtle forms of emotional adjustment. In Case 19, for example, the participant displayed a degree of social composure rarely observed in other compliance-oriented hearings. He sat upright, maintained a calm demeanor, and responded in a measured and organized manner. The prosecutor, in turn, avoided unnecessary probing and moderated the intensity of questioning. Brief moments of eye contact were followed by mutual withdrawal of gaze, producing a tacit form of interpersonal accommodation. Neither side openly acknowledged this adjustment, yet posture, tone, and pacing collectively contributed to a smooth interaction. Emotional negotiation remained limited, but a degree of situational coordination emerged through nonverbal interaction.

Case 20 represents a further variation. Many years after the original incident, the participant reflected on his earlier wrongdoing calmly and thoughtfully. His speech occasionally slowed as he revisited past experiences, and he sometimes looked toward the hearing officers with a quiet expression of sincerity. One hearing officer gradually set aside the case materials and listened attentively, responding with slight nods and visible signs of concern. Although the hearing remained procedurally routine and no substantive emotional negotiation occurred, a modest degree of affective empathy emerged through attentive listening and acknowledgment. This moment of interpersonal resonance did not fundamentally alter the interactional pattern of the hearing, but it demonstrated that even within a generally low-emotion environment, a limited emotional connection could occasionally develop.

### The gladiatorial confrontation pattern

The gladiatorial confrontation pattern is characterized by high emotional arousal, confrontational forms of emotional expression, sustained emotional negotiation, and the absence of affective empathy. Hearings displaying this pattern commonly involved disputes over compensation, procedural legitimacy, personal injury, civil enforcement, or other matters in which participants perceived significant interests to be at stake. Participants often entered the hearing carrying accumulated feelings of grievance, frustration, or perceived injustice. Unlike the compliance pattern, emotional tension was already present before the formal hearing began and remained a defining feature of the interaction throughout the proceedings.

Participants’ emotional arousal was visible from the outset. Upon entering the hearing room, many adopted rigid postures, leaned forward in their seats, or maintained prolonged eye contact with prosecutors. Their attention was directed less toward procedural completion than toward defending their own interpretations of events. Emotional investment in the hearing outcome was often evident before substantive discussion had even begun. Facial expressions frequently conveyed dissatisfaction, suspicion, or determination. In several cases, participants appeared prepared for disagreement prior to the first formal question being asked.

These heightened emotional states became visible through direct and expressive forms of emotional expression. Participants often responded immediately when hearing statements they considered unfavorable. Speech accelerated, voices became louder, and hand gestures became more animated. Some interrupted prosecutors before questions were completed, while others repeatedly returned to particular grievances despite attempts to redirect the discussion. Table tapping, emphatic pointing, prolonged staring, and repeated challenges to procedural decisions functioned as visible expressions of frustration and resistance. Unlike the restrained responses observed in compliance-oriented hearings, emotions were openly displayed and actively communicated to others.

The most distinctive feature of this interactional pattern was the emergence of sustained emotional negotiation. Emotional expressions did not remain individual reactions but became resources through which participants attempted to influence one another. Challenges from participants prompted responses from prosecutors, whose replies in turn generated further reactions. Interaction unfolded as a continuous process of emotional assessment and adjustment. Participants monitored how their expressions affected others, while prosecutors evaluated when to insist, when to explain, and when to avoid further escalation. The atmosphere of the hearing was therefore not fixed but continuously shaped through reciprocal emotional exchanges.

This confrontational dynamic was clearly reflected in participants’ verbal practices. In Case 2, the participant repeatedly interrupted the prosecutor and challenged the legal characterization of her conduct: *“I was not driving on a public road!”; “I do not accept this determination!”; “I will not respond to what you said about the blood alcohol level. What can this number actually prove?”; “Some people drink only a small amount but have a very high reading, while others drink an entire bottle of red wine and the reading is still not high!”* Her voice gradually became louder, and she expressed suspicion of what she perceived as entrapment by law enforcement: *“I strongly suspect that the traffic police and the designated driver company worked together to deceive me.”* In Case 10, the participant emotionally questioned the court enforcement procedure: *“I never received anything. My account was frozen for no reason. How could the court handle a case so carelessly? How can people still believe that the law is fair?”* When advised to calm down, she responded: *“I cannot calm down. This is such a simple matter. It could be clarified as long as someone seriously investigates it.”* In Case 16, after listening to the bank representative’s statement, the participant angrily accused the bank of being shameless and going back on its word, accompanied by table tapping. In Case 17, the participant threatened further escalation: *“If I do not receive a satisfactory response today, I will submit a petition to the authorities.”* These verbal practices, including interruptions, direct challenges, increased vocal intensity, derogatory accusations, and threats of petitioning, functioned as discursive resources through which participants articulated their positions and sustained emotional negotiation.

The aforementioned interruptions, challenges, increased vocal intensity, and derogatory accusations were coded as confrontational emotional expression, while the reciprocal exchanges between prosecutors and participants were coded as sustained emotional negotiation. Emotional negotiation in this pattern unfolded in a competitive rather than conciliatory manner and therefore failed to generate affective empathy.

Prosecutors occupied a particularly important position within this process. Although they generally maintained controlled and professional demeanors, they were actively involved in emotional negotiation. When confronted with anger or accusations, prosecutors rarely responded with equivalent emotional intensity. Instead, they typically relied on procedural explanations, legal reasoning, and measured language to re-establish interactional order. At times, however, they adjusted their pace of speech, reformulated questions, or introduced clarifying narratives in an effort to prevent further escalation. These responses did not eliminate conflict but influenced how it unfolded during the hearing. Emotional negotiation, therefore, involved not only disagreement over substantive issues but also ongoing efforts to manage the emotional direction of the interaction itself.

Hearing officers were also drawn into these dynamics, although their role remained largely reactive. As exchanges intensified, hearing officers frequently shifted their attention between the opposing sides, closely monitoring both verbal content and emotional tone. Their facial expressions often revealed concern or uncertainty. Some appeared ready to intervene but hesitated when opportunities arose, unsure whether intervention would calm or intensify the situation. Rather than remaining detached observers, they became emotionally affected by the interaction while possessing only a limited capacity to reshape it. Their participation illustrates how emotional atmospheres influence institutional actors beyond the immediate disputing parties.

Cases 2, 10, 13, 16, 17, and 18 most clearly reflect this interactional pattern. In one dangerous-driving hearing, the participant immediately challenged the prosecutor’s characterization of responsibility, raising his voice and repeatedly questioning the factual basis of the decision. Throughout the exchange, he maintained direct eye contact and frequently interrupted attempts to move to subsequent procedural issues. In a civil enforcement hearing, another participant repeatedly returned to the freezing of a bank account, describing the measure as unfair and disproportionate. Although prosecutors patiently explained procedural requirements and legal justifications, neither side substantially modified its position. What emerged was not a dialogue aimed at consensus but an extended process of mutual challenge and response.

Periods of silence also carried distinctive meanings within this pattern. Unlike the compliance hearings, where silence often reflected passivity or acceptance, pauses in confrontation-oriented hearings frequently emerged after particularly contentious exchanges. During these moments, participants remained visibly tense, maintaining rigid posture or fixed gaze while waiting for the other side to respond. Silence functioned less as withdrawal than as a temporary suspension of open disagreement. The underlying conflict remained unresolved and quickly resurfaced once interaction resumed.

Affective empathy rarely emerged within this interactional environment. Participants remained focused on defending their own positions and interpreting the actions of others through adversarial lenses. Although prosecutors occasionally acknowledged participants’ frustrations, such acknowledgements seldom developed into deeper emotional understanding. Likewise, participants rarely demonstrated receptiveness toward the institutional constraints faced by prosecutors. Emotional negotiation therefore remained primarily competitive rather than conciliatory. The interaction generated emotional energy and sustained engagement, but it did not produce mutual emotional resonance.

Case 5 illustrates an important variation within this broader pattern. The participant entered the hearing displaying visible dissatisfaction and appeared prepared for confrontation. Anticipating escalation, however, the prosecutor adopted a different interactional strategy. Rather than responding through procedural insistence alone, the prosecutor deliberately slowed the pace of the conversation, encouraged the participant to speak uninterrupted, and acknowledged concerns before presenting legal explanations. The prosecutor’s posture became noticeably less formal, and moments of attentive listening replaced rapid question-and-answer exchanges. As the hearing progressed, the participant’s speech gradually slowed, interruptions became less frequent, and visible tension eased. Although full affective empathy did not emerge, emotional negotiation shifted from competitive confrontation toward cautious accommodation. This case demonstrates that confrontation-oriented hearings are not fixed interactional states. Their trajectory depends on how participants respond to one another’s emotional expressions and whether emotional negotiation succeeds in reducing or reproducing tension.

### The negotiated empathy pattern

The negotiated empathy pattern is characterized by moderate to high emotional arousal, narrative forms of emotional expression, flexible emotional negotiation, and the emergence of affective empathy among participants. Hearings displaying this pattern commonly involved community corrections, parole assessments, public-interest dispute resolution, and cases associated with rehabilitation, family responsibility, or personal remorse. Participants often entered the hearing carrying considerable emotional burdens arising from past experiences, financial pressures, caregiving responsibilities, or concern about prospects. Unlike the confrontation pattern, however, these emotions were rarely directed toward challenging others. Instead, they became resources through which participants communicated personal circumstances and sought understanding.

Emotional arousal remained clearly present throughout these hearings, although it was expressed differently from the anger and grievance observed in confrontation-oriented cases. Participants frequently described feelings of regret, anxiety, responsibility, uncertainty, or concern for family members. These emotions were often linked to broader life circumstances rather than immediate disputes over legal outcomes. As a result, emotional intensity did not generate opposition but instead created opportunities for personal disclosure. Participants entered hearings not merely as legal subjects responding to procedural questions but as individuals carrying complex emotional experiences shaped by their everyday lives.

The dominant form of emotional expression within this pattern was narrative expression. Rather than offering brief procedural answers, participants frequently recounted personal histories, family situations, employment difficulties, rehabilitation experiences, or reflections on past mistakes. These narratives unfolded gradually and often contained emotionally significant details that extended beyond the formal legal issues under review. Participants typically spoke in calm and measured tones, occasionally pausing to collect their thoughts before continuing. Their accounts rarely appeared strategic or exaggerated. Instead, they conveyed a sense of sincerity and vulnerability that encouraged attentive listening from others.

Emotional negotiation played a crucial role in shaping the interactional atmosphere of these hearings. Unlike the adversarial exchanges observed in confrontation-oriented cases, emotional negotiation here was primarily oriented toward accommodation and mutual understanding. Prosecutors actively adjusted the pace of questioning in response to participants’ emotional cues. Follow-up questions often invited further elaboration rather than seeking contradiction or clarification. Participants responded positively to these opportunities, expanding upon their experiences and becoming increasingly comfortable sharing personal concerns. Through this reciprocal process, emotional expression and emotional response gradually became aligned.

Narrative expression within this pattern was achieved through extended personal narratives rather than brief procedural responses. In Case 8, when asked why he had left his residential area without authorization, the participant did not simply deny wrongdoing. Instead, he provided a family-centered account: *“Both of my children are still young. The older one is twelve, and the younger one is in the final year of kindergarten. I have to support both of them.”* His voice became choked with emotion, and his body trembled slightly. The prosecutor first acknowledged his circumstances before asking for further explanation: *“I can understand your situation, but objectively speaking, you did violate the supervision requirements. Why did you not report the situation to the judicial office beforehand?”* In Case 12, when asked about his views on parole, the participant expressed gratitude and remorse: *“I am truly grateful that the government has given me this opportunity.” “I did make mistakes, and I have been trying to correct them through my actions. If I am given the opportunity to be released early, I will definitely cherish it.”* When mentioning his daughter, his voice became choked again. The hearing officer subsequently inquired about his family support system, inviting him to further express his emotions. In Case 9, two of the three participants expressed remorse through direct and emotionally charged statements: *“I was truly wrong. Please give me another chance,”* and *“I was really wrong. I will never do this again.”* These expressions stood in sharp contrast to the silence and sarcastic laughter displayed by the third participant, Deng. These verbal and interactional features, including personal narratives, affective vocabulary, pauses and emotional choking, prosecutors’ accommodative follow-up questions, and hearing officers’ supportive inquiries, collectively created the conditions for the emergence of affective empathy.

The participants’ family-centered narratives were coded as indicators of emotional arousal and narrative emotional expression; prosecutors’ accommodative follow-up questions were coded as adaptive adjustments within emotional negotiation; and hearing officers’ supportive inquiries were coded as manifestations of affective empathy. The combination of these three dimensions constituted the typical configuration of the negotiated empathy pattern.

The hearing atmosphere was therefore characterized by a slower and more flexible interactional rhythm. Long pauses were not interpreted as resistance or hesitation but as opportunities for reflection. Participants were rarely interrupted while speaking, and conversational exchanges often extended beyond the minimum procedural requirements. Prosecutors frequently acknowledged participants’ experiences through supportive language, gentle questioning, and attentive listening. Nonverbal communication also played an important role. Slight nods, relaxed posture, sustained eye contact, and patient silence communicated recognition and respect, helping to reinforce a sense of interpersonal trust within the hearing environment.

Hearing officers were noticeably more involved in these hearings than in the other interactional patterns. Rather than remaining passive observers, they frequently became active participants in the emotional flow of the interaction. They listened attentively to participants’ narratives, maintained sustained visual engagement, and asked questions concerning family circumstances, rehabilitation efforts, or plans. Their interventions often reflected curiosity, concern, and encouragement rather than procedural oversight alone. Through these interactions, hearing officers contributed to an atmosphere in which participants felt heard and understood.

Cases 3, 7, 9, 11, 12, and 14 most clearly illustrate this interactional pattern. Across these hearings, participants gradually moved beyond procedural discussion and shared experiences that revealed the broader human context surrounding their cases. Narratives of family responsibility, personal growth, economic hardship, and efforts at rehabilitation became central components of the interaction. Prosecutors frequently adjusted the structure of questioning to accommodate these accounts, while hearing officers responded with visible attentiveness and concern. Deliberative discussions likewise moved beyond formulaic legal assessments and incorporated consideration of participants’ lived circumstances. Rather than defending competing positions, participants collectively worked toward a shared understanding of the issues being discussed.

The emergence of affective empathy distinguished this pattern most clearly from the other forms of hearing interaction. Empathy did not arise suddenly or automatically. Instead, it developed gradually through sustained cycles of emotional expression and emotional negotiation. As participants shared personal experiences and others responded attentively, emotional distance narrowed. Prosecutors became more receptive to participants’ circumstances, hearing officers expressed greater understanding of personal difficulties, and participants themselves appeared increasingly willing to engage openly with institutional actors. Emotional resonance emerged not because formal roles disappeared but because participants became temporarily capable of seeing beyond those roles.

Case 8 provides a particularly clear illustration of this process. The participant, who was responsible for supporting an entire family while undergoing community correction, described daily pressures and family responsibilities in a subdued and reflective manner. His speech was slow and deliberate, and moments of silence occasionally accompanied particularly difficult topics. Throughout the account, the prosecutor refrained from interruption and allowed the narrative to unfold at its own pace. Nearby hearing officers gradually leaned forward, maintaining focused attention and displaying visible concern through facial expressions and sustained eye contact. After the participant finished speaking, responses were delivered in a gentle and encouraging tone. Questions focused not only on compliance requirements but also on plans and family well-being. No intense disagreement emerged, nor was there any effort to accelerate the hearing process. Instead, emotional understanding developed through attentive listening, narrative sharing, and supportive interaction. Through this process, a routine procedural hearing acquired a distinctly human dimension, demonstrating how affective empathy can emerge within formal legal settings and reshape the character of interaction itself.

### Hearing officers as responsive participants across interactional patterns

Across the 20 hearings examined in this study, hearing officers did not occupy a fixed interactional role. Although their formal responsibilities remained identical across cases, their actual level of participation varied considerably. These variations cannot be adequately explained by institutional rules or procedural requirements, which remained largely unchanged across hearings. Instead, field observations suggest that hearing officers’ involvement was closely associated with the emotional dynamics unfolding during interaction. Their participation emerged as a response to different configurations of emotional arousal, emotional expression, emotional negotiation, and affective empathy.

In hearings characterized by the assembly-line compliance pattern, hearing officers remained largely detached from the interactional process. The combination of low emotional arousal, restrained emotional expression, and limited emotional negotiation provided few opportunities or incentives for active involvement. Most hearing officers concentrated on reviewing case materials and monitoring procedural progress. Their attention was directed primarily toward factual and procedural matters rather than participants’ emotional experiences. Eye contact with parties was infrequent, interventions were rare, and communication among hearing officers remained minimal. Because emotional concerns were seldom openly expressed, hearing officers had little reason to move beyond their procedural role.

A different pattern emerged in confrontation-oriented hearings. Here, hearing officers became increasingly attentive to the interaction as emotional intensity escalated. Strong emotional expressions, repeated challenges, and sustained emotional negotiation drew them into the unfolding exchanges. Their gaze frequently shifted between participants, and their facial expressions often reflected concern, uncertainty, or anticipation. Yet heightened attention did not necessarily translate into active intervention. Many appeared aware of the tension but remained hesitant to interrupt the interaction. Entering the exchange risked being interpreted as taking sides, potentially intensifying rather than reducing conflict. As a result, hearing officers often occupied a position of emotionally engaged observation. They became participants in the emotional atmosphere without becoming active participants in the negotiation itself.

The negotiated empathy pattern generated a markedly different form of involvement. As participants shared personal experiences and emotional concerns, hearing officers became progressively integrated into the interactional process. Narrative forms of emotional expression provided opportunities for engagement, while accommodation-oriented emotional negotiation reduced the risks associated with intervention. Hearing officers, therefore, participated more actively through attentive listening, supportive questioning, and expressions of concern. Their contributions were not directed toward resolving legal disagreements but toward sustaining an atmosphere conducive to communication and understanding. Under these conditions, hearing officers became important participants in the production and maintenance of affective empathy.

The verbal practices of hearing officers reflected these differences. Within the negotiated empathy pattern, hearing officers’ questions frequently shifted from procedural oversight toward personal concerns. For example, in Case 8, hearing officers inquired about the participant’s family circumstances and employment arrangements; in Case 12, one hearing officer asked, *“What do your family members think about this matter?”* thereby inviting the participant to further elaborate on sources of family support. In contrast, within the confrontation-oriented pattern, hearing officers generally remained silent or intervened only at procedural points. In Case 10, for instance, hearing officers primarily focused on reviewing evidentiary materials while the participant expressed anger, whereas in Case 17, they observed the dispute between the two sides without actively raising questions. Within the assembly-line compliance pattern, hearing officers’ questions were relatively limited and largely procedural in nature. In Case 1, for example, their inquiries mainly focused on confirming the juvenile participant’s school enrollment and developmental records.

Supportive questioning by hearing officers in the negotiated empathy pattern was coded as affective empathy; silence or procedural intervention in confrontation-oriented hearings was coded as limited emotional negotiation; and fact-oriented questioning in assembly-line compliance hearings was coded as procedural detachment. These coding results indicate that variations in hearing officers’ participation were closely associated with configurations of emotional dynamics rather than being determined solely by their institutional roles.

Several atypical cases further illustrate the responsiveness of hearing officers’ participation to interactional dynamics rather than to institutional role expectations alone. In Case 20, despite the overall compliance-oriented character of the hearing, one hearing officer gradually shifted from reviewing documents to listening closely as the participant reflected on past mistakes and personal growth. Through sustained attention, nodding, and visible concern, a modest form of empathic engagement emerged. Conversely, in Case 5, hearing officers remained relatively passive even as a potentially confrontational interaction was successfully redirected toward accommodation. Because the prosecutor assumed primary responsibility for emotional regulation, hearing officers had little need to intervene directly. These cases suggest that hearing officers’ participation is not mechanically determined by hearing type but is shaped by how emotional interactions unfold in specific situations.

These observations indicate that hearing officers function less as fixed institutional actors than as responsive participants whose involvement fluctuates in relation to the emotional dynamics of hearings. Their participation increases when emotional expression invites engagement, when emotional negotiation creates opportunities for intervention, and when affective empathy becomes possible. Conversely, participation remains limited when emotional interactions are either absent or highly polarized. Variations in hearing officers’ behavior therefore reflect broader interactional processes rather than individual differences in personality or commitment.

This finding helps explain why hearing practices may diverge despite operating within a common procedural framework. The formal structure of hearings establishes the institutional setting within which interaction occurs, but it does not fully determine how participants engage with one another. Hearing officers, prosecutors, and parties continuously respond to emotional cues generated during interaction, and these responses contribute to the development of different hearing trajectories. From this perspective, variations in hearing officers’ participation are not secondary features of hearing practice but constitute an important indicator of the underlying emotional dynamics through which distinct forms of hearing interaction emerge.

## Discussion

Drawing on immersive field observations of 20 grassroots prosecutorial hearings, this study explains how diverse forms of hearing practice emerge within a formally standardized procedural framework. The findings demonstrate that procedural rules alone do not determine how hearings unfold in practice. Although all hearings observed in this study followed the same institutional procedures, they developed into three relatively stable interactional patterns: assembly-line compliance, gladiatorial confrontation, and negotiated empathy. These patterns differed not primarily because of variations in legal procedures or organizational arrangements, but because of distinct configurations of emotional arousal, emotional expression, emotional negotiation, and affective empathy. The findings therefore suggest that emotional interaction constitutes an important mechanism through which institutional uniformity coexists with practical diversity in grassroots prosecutorial hearings.

This conclusion resonates with growing international scholarship emphasizing the significance of emotion in legal practice. Research on legal professions has demonstrated that emotion management, interpersonal empathy, and emotional interaction are integral components of judicial work rather than deviations from legal rationality (Bergman Blix and Wettergren, 2019). Evidence from the Dutch judicial context similarly indicates that emotional narratives can be incorporated into legal proceedings without compromising professional objectivity, as legal actors routinely engage in emotional labor while maintaining procedural impartiality (Bosma, 2024). Existing studies have also highlighted the importance of both cognitive and affective dimensions of empathy in improving legal communication and decision-making processes (Westaby and Jones, 2018). The present study extends these insights by showing how emotional processes do not merely accompany hearings but actively shape the emergence of different forms of hearing interaction.

More broadly, the study contributes to research on judicial rituals by introducing an emotion-centered explanation for variation in hearing practices. Existing scholarship has often explained judicial rituals through concepts such as institutional authority, symbolic performance, bureaucratic organization, and power relations. These perspectives have been highly valuable for understanding how legal institutions establish legitimacy and maintain procedural order. Research in the Chinese context has similarly demonstrated how tensions between formal institutions and local social realities may reshape the practical enactment of judicial rituals (Su, 2000), while participants’ interactions can influence the implementation and effectiveness of legal procedures (Li, 2021). Interaction ritual chain theory further identifies emotional energy as a central force underlying ritual processes and collective interaction (Collins, 2004). Building upon these insights, the present study shifts analytical attention from institutional structures alone to the emotional dynamics through which judicial order is continuously produced, reproduced, and transformed during interaction. The findings suggest that different ritual forms emerge not simply because participants occupy different institutional positions, but because they engage in distinct emotional processes that shape the trajectory of interaction.

A central theoretical contribution of this study lies in the development and empirical application of a four-dimensional framework consisting of emotional arousal, emotional expression, emotional negotiation, and affective empathy. Rather than treating emotions as isolated psychological states, the findings suggest that emotions operate as a sequential and relational interactional process. Emotional arousal provides the initial emotional conditions that participants bring into hearings, shaped by prior experiences, perceived interests, personal circumstances, and expectations regarding legal outcomes. These emotional states subsequently become observable through emotional expression, which takes different forms across hearing contexts, including restrained expression, confrontational expression, and narrative expression. Emotional expression therefore functions as the primary mechanism through which internal emotional experiences become socially visible within interaction. By decomposing emotional dynamics into four interconnected dimensions and specifying observable interactional indicators, the analytical framework developed in this study provides a systematic operational pathway for comparing and explaining judicial interactions while maintaining qualitative sensitivity.

It should be emphasized that the identification of the four emotional dimensions in this study was not based on abstract inference, but on a systematic analysis of participants’ discursive practices. Emotional arousal was identified through the content of participants’ narratives and their use of affective vocabulary. Emotional expression was captured through interactional features such as response length, vocal intensity, interruptions, silence, and narrative styles. Emotional negotiation was examined through dynamic adjustments in the questioning and response patterns among prosecutors, parties, and hearing officers. The emergence of affective empathy was identified through shifts from procedural questioning to supportive inquiries and from factual statements to personal narratives. This discourse-sensitive approach provides an empirical foundation for the analytical framework while remaining consistent with the study’s abductive thematic analysis methodology.

Among the four dimensions, emotional negotiation emerges as the most important mechanism linking individual emotional experiences to collective interactional outcomes. Unlike emotional arousal and emotional expression, which focus primarily on individual emotional conditions and their manifestation, emotional negotiation captures the dynamic process through which participants respond to, adjust to, and strategically engage with one another’s emotional displays. The concept emerged inductively from the field observations and proved essential for explaining why similar emotional conditions sometimes generated very different hearing trajectories. Across the hearings, participants continuously interpreted emotional cues, modified their communicative strategies, and adjusted their responses in relation to others. Whether hearings evolved toward procedural compliance, sustained confrontation, or mutual understanding depended largely on how emotional negotiation unfolded during interaction. Emotional negotiation therefore serves as the critical mechanism connecting individual emotions with broader patterns of hearing practice.

The findings also demonstrate that affective empathy represents neither an automatic consequence of emotional expression nor a universal outcome of emotional negotiation. Rather, affective empathy emerges only under specific interactional conditions in which emotional negotiation facilitates mutual recognition and emotional receptivity. In the negotiated empathy pattern, participants gradually moved beyond formal role expectations and became responsive to one another’s experiences and circumstances. By contrast, in confrontation-oriented hearings, emotional negotiation often remained competitive rather than accommodative, preventing the emergence of empathic engagement. These observations suggest that hearing trajectories are shaped not by isolated emotional factors but by the evolving configuration of the four interconnected dimensions. The framework therefore offers a parsimonious explanation for how diverse forms of hearing interaction emerge under a common procedural structure.

The study also contributes to the expanding literature on emotion in judicial settings. Existing research has examined emotional processes across a variety of legal contexts, including courtroom adjudication (Johansen et al., 2023) and community mediation (Li and Liu, 2025). In practice, emotional support and emotional management have increasingly been incorporated into community correction policies and professional guidelines [Ministry of Justice of the People’s Republic of China (MoJ), 2021]. These studies have established an important foundation for understanding emotion as a significant component of legal interaction. However, systematic examination of emotional processes within prosecutorial hearings remains limited. Given the hybrid institutional character of prosecutorial hearings, which combine judicial and administrative functions while accommodating both legal reasoning and contextual considerations, emotional dynamics assume particular importance. By examining emotional interaction within this distinctive institutional setting, the present study extends the scope of judicial emotion research and demonstrates how governance outcomes such as conflict resolution, relationship repair, and procedural acceptance emerge through observable interactional processes rather than through procedural design alone.

A second contribution concerns the role of hearing officers. As a localized institutional arrangement in China, prosecutorial hearings have attracted limited empirical attention regarding the practical role of hearing officers. Existing studies primarily focus on legislative design, institutional reform, and procedural optimization, while the small body of research directly examining hearing officers often relies on role theory (Biddle, 1979) or dramaturgical perspectives (Goffman, 1959) to define their expected institutional functions (Lv and Fu, 2026). Such approaches help clarify formal role expectations but provide limited insight into how hearing officers actually participate in hearings under different interactional conditions.

Drawing on field observations, this study suggests that hearing officers function less as fixed institutional actors than as responsive participants whose involvement varies according to emotional dynamics. Across the three hearing patterns, hearing officers moved along a continuum ranging from procedural detachment to emotionally engaged observation to active empathic participation. Their level of involvement was shaped not primarily by institutional status or formal responsibilities, but by the interactional configuration of emotional arousal, emotional expression, emotional negotiation, and affective empathy unfolding during the hearing itself. This finding shifts analytical attention from static role definitions toward the dynamic processes through which institutional roles are enacted, adjusted, and transformed in practice. It also demonstrates that variations in hearing officers’ participation reflect broader emotional processes operating within judicial interactions.

Finally, the study sheds light on the relationship between institutional standardization and practical variation in grassroots judicial governance. Existing discussions of prosecutorial hearing reform have largely focused on procedural refinement, institutional optimization, and regulatory improvement. While such efforts contribute to procedural consistency, they provide only a partial explanation for the diversity of hearing practices observed in local contexts. The findings suggest that variation should not be understood simply as a deviation from institutional design. Rather, different hearing forms represent adaptive interactional responses to distinct emotional conditions and case circumstances. This perspective implies that future efforts to improve prosecutorial hearings may benefit not only from procedural safeguards and institutional reforms, but also from greater attention to emotional dynamics within legal interaction. Constructive emotional arousal, appropriate emotional regulation, flexible emotional negotiation, and the cultivation of affective empathy may contribute to more productive hearing environments and facilitate the effective realization of the governance functions of prosecutorial hearings in practice.

## Conclusion

This study examines how grassroots prosecutorial hearings become differentiated within a formally standardized procedural framework. Drawing on immersive field observations of 20 hearings, it shows that hearing practices cannot be fully explained by procedural arrangements alone. Instead, different interactional patterns emerge through the configuration of emotional arousal, emotional expression, emotional negotiation, and affective empathy. By identifying three relatively stable forms of hearing interaction and accounting for atypical cases within the same analytical framework, the study demonstrates that emotional dynamics constitute an important mechanism shaping the practical operation of grassroots prosecutorial hearings.

The study contributes to existing research by developing an emotion-centered perspective for understanding judicial interaction. Rather than focusing primarily on institutional structures or symbolic functions, it highlights how emotional processes influence participants’ engagement, interactional trajectories, and hearing outcomes. The findings suggest that the effectiveness of prosecutorial hearings depends not only on procedural design but also on the quality of emotional interaction among participants. Greater attention to emotional communication, flexible negotiation, and empathic engagement may therefore contribute to more constructive hearing practices and improve the governance functions of grassroots prosecutorial hearings.

Two limitations should be acknowledged. First, the study is based on fieldwork conducted across eight district-level procuratorates within a single provincial capital city. Although this design captures variation across multiple district jurisdictions within the city, the findings should be interpreted with caution and cannot be assumed to represent hearing practices in other cities or regions. Second, the analysis focuses primarily on emotional interaction and does not systematically examine broader institutional and organizational influences. Future research could test the applicability of the proposed framework across different jurisdictions, judicial settings, and online hearing environments, while further exploring the relationship between emotional dynamics and institutional conditions.

## Data Availability

The original contributions presented in the study are included in the article/supplementary material, further inquiries can be directed to the corresponding author.
