# Supplementary material for: Emotional dynamics and hearing differentiation under a unified procedure: a field study of grassroots procuratorial hearings in China
Source: Front Psychol. 2026 Aug 26;17:1912074. doi: 10.3389/fpsyg.2026.1912074 (PMC13553336; doi:10.3389/fpsyg.2026.1912074)
Supplement: Supplementary file 1 [file Supplementary_file_1.docx]

**Appendix A: Case Overview**

The twenty cases included in this appendix cover a wide range of case types, including juvenile offenses, dangerous driving, food safety violations, civil enforcement supervision, community correction, parole, self-defense, and public interest litigation. These cases reflect the broad applicability of procuratorial hearings within grassroots judicial practices in China. The case summaries presented in this appendix were recorded through the first author’s on-site observations in the capacity of a hearing officer and were independently reviewed and interpreted by the second and third authors. The case summaries are provided solely for contextual background. Each case is analyzed in the main text according to the four-dimensional analytical framework described in the Methodology section, and the specific definitions of each dimension and analytical procedures are presented therein.

**Case 1 (District A): Juvenile Affray**

Five juveniles aged 16-18 became involved in a group fight following a personal dispute in a residential complex, during which one participant carried a controlled knife, and the group chased and assaulted several peers, causing minor injuries. At the closed hearing attended by guardians, teachers, and legal representatives, prosecutors presented surveillance evidence and emphasized both the seriousness of the violence and the juveniles’ post-arrest cooperation. The participants showed mixed responses ranging from remorse to instrumental reliance on their juvenile status. The discussion ultimately focused on educational correction and structured supervision rather than punitive escalation, and a conditional non-prosecution decision with a six-month supervision period was recommended.

**Case 2 (District B): Drink Driving Case**

A female driver consumed alcohol at a dinner gathering and later drove within a residential compound after initially using a designated driver, where she was tested with a blood alcohol level exceeding the legal limit. During the hearing, she first disputed the legal characterization of her conduct, arguing that she had not driven on public roads, but gradually shifted to acceptance after prosecutors clarified legal standards and evidentiary requirements. The hearing emphasized clarification and persuasion rather than confrontation, and participants ultimately reached consensus through interpretive alignment, resulting in relative non-prosecution.

**Case 3 (District G): Toxic Food Production**

A food processing enterprise was found to have illegally added prohibited substances to enhance product quality, resulting in serious food safety risks identified through regulatory sampling. At an expert-involved hearing, legal scholars and practitioners expressed sharply divergent views between strict punitive deterrence and proportional enforcement feasibility. The discussion centered on balancing legal severity with practical implementation constraints, and the outcome reflected institutional compromise, combining criminal prosecution with civil public interest litigation.

**Case 4 (District C): Juvenile Theft Series**

Several juveniles repeatedly stole low-value goods such as cigarettes and beverages from convenience stores over an extended period, cumulatively reaching criminal thresholds despite individually minor amounts. At the closed hearing, participants displayed generally indifferent attitudes, with limited emotional engagement or moral reflection and repeated minimization of wrongdoing. The process revealed procedural participation without substantive internalization, reflecting a passive compliance pattern, and conditional non-prosecution with mandatory supervision was imposed.

**Case 5 (District A): Neighbor Assault**

A long-standing neighborhood dispute escalated into physical violence when the suspect struck the victim with a wooden stick during an argument over shared residential space, causing minor injuries, although substantial compensation had already been paid before the hearing. During the hearing, the victim remained silent while the suspect emphasized provocation and long-term conflict history. The discussion gradually shifted toward reconciliation and compensation adequacy rather than strict culpability assessment, and a mediated resolution led to relative non-prosecution.

**Case 6 (District F): Assistance to Cyber Fraud**

A university student provided personal bank and phone cards to others for profit under a so-called part-time arrangement, which were later used in large-scale telecom fraud involving multiple victims and significant losses. At the hearing, prosecutors highlighted downstream harm, and the student initially appeared emotionally unstable and highly remorseful. After being informed that victims had accepted compensation and forgiveness, he showed a strong emotional release and full acceptance of responsibility, resulting in a compliance-oriented resolution and relative non-prosecution.

**Case 7 (District D): Bail Travel Permission**

A business owner under bail requested permission to travel outside the jurisdiction to finalize a major commercial contract affecting employment for dozens of workers. Prosecutors raised concerns about supervision risk and flight possibility, while the suspect provided detailed assurances of compliance and necessity. The hearing evolved into a structured risk-benefit evaluation rather than a binary approval process, and conditional permission was granted with strict reporting obligations.

**Case 8 (District A): Community Supervision Violation**

A probationer left the jurisdiction for work without authorization and failed to report his whereabouts for several months, constituting a formal violation of supervision requirements. At the hearing, he admitted the factual conduct but framed it primarily as an economic necessity and showed limited reflection on legal obligations. The discussion emphasized insufficient behavioral transformation, reflecting passive compliance, and a formal warning with continued supervision was issued.

**Case 9 (District E): Exam Cheating**

Three examinees used electronic devices to obtain answers during a national examination and were subsequently detected by invigilators. At the hearing, two individuals admitted wrongdoing with remorse, while one persistently refused cooperation and rejected responsibility throughout questioning. This created a clear divergence between compliance-oriented acceptance and resistance-based denial, leading to conditional non-prosecution for two individuals and prosecution for the non-cooperative suspect.

**Case 10 (District F): Civil Enforcement Supervision**

A debtor challenged court enforcement measures involving the freezing of company shares in a loan dispute, arguing partial repayment arrangements had not been fully considered, while enforcement authorities maintained procedural legality. During the hearing, both sides presented legal arguments, but the discussion gradually shifted toward settlement feasibility and repayment structuring. The process reflected pragmatic mediation, and the legality was upheld alongside a negotiated repayment agreement.

**Case 11 (District A): Probation Violation**

A probationer repeatedly violated supervision obligations, failed to report for extended periods, and demonstrated minimal engagement with correctional requirements. At the hearing, he returned but minimized responsibility and challenged the seriousness of his violations, showing persistent non-cooperation. The proceedings reflected escalating resistance toward correctional authority, and revocation of probation with enforcement of the original sentence was recommended.

**Case 12 (District A): Parole**

A long-term prisoner demonstrated consistent disciplinary adherence, active participation in rehabilitation programs, and stable behavioral improvement throughout incarceration. At the hearing, correctional authorities and stakeholders uniformly confirmed his reform trajectory, and he expressed structured remorse and responsibility awareness. The process reflected full institutional alignment, and parole was approved.

**Case 13 (District D): Self-defense Case**

A suspect injured one of several attackers during a violent confrontation in which he was pursued and assaulted by multiple individuals. The hearing focused on whether defensive conduct exceeded necessary limits, with close examination of evidence and situational escalation. The discussion emphasized proportionality and necessity rather than moral judgment, and the conduct was recognized as legitimate self-defence with no prosecution.

**Case 14 (District B): Medical Parole**

A prisoner suffering from severe chronic illness requiring continuous treatment beyond prison medical capacity was evaluated for temporary release. At the hearing, medical experts, correctional officials, and family members jointly assessed health conditions and supervision risks. The process balanced humanitarian necessity with correctional security considerations, resulting in approval of temporary medical parole under supervision.

**Case 15 (District G): Administrative Supervision Recommendation**

A local administrative office failed to effectively address sanitation and fire safety hazards in aging residential areas despite repeated prosecutorial warnings. At the hearing, officials acknowledged resource constraints while residents described ongoing risks to daily life and safety. The discussion focused on governance capacity and corrective planning, resulting in a supervisory recommendation to enforce institutional accountability.

**Case 16 (District E): Loan Enforcement Dispute**

A rural borrower challenged bank enforcement actions concerning interest, penalties, and compound interest, citing financial hardship and contractual imbalance. During the hearing, the bank defended contractual legality while the borrower expressed willingness to repay the principal through instalments. The process combined legal reaffirmation with settlement-oriented negotiation, resulting in upheld enforcement and a structured repayment agreement.

**Case 17 (District F): Property Contract Dispute**

A property seller refused to complete the ownership transfer after receiving partial payment, citing disputes over remaining obligations and alleged defects, while the buyer insisted on contractual enforcement. At the hearing, both parties maintained legal positions but gradually moved toward settlement discussions. The process reflected mediation-driven pragmatism, resulting in a settlement agreement alongside confirmation of the court judgment.

**Case 18 (District H): Group Assault**

Five individuals participated in a violent group assault at a night market, causing serious injuries to one victim and minor injuries to another. At the hearing, all suspects confessed and expressed remorse, while victims confirmed compensation and requested leniency. The proceedings reflected coordinated acceptance of legal responsibility with mitigation considerations, and prosecution with sentencing recommendations was adopted.

**Case 19 (District B): Smuggling Case**

A suspect engaged in repeated smuggling of luxury goods for profit through cross-border transport and online resale channels. After the investigation, he voluntarily confessed, returned illicit gains, and fully cooperated throughout proceedings. The hearing showed no factual dispute and strong procedural alignment, reflecting full compliance, and a suspended sentence with a fine was recommended.

**Case 20 (District H): Robbery Case**

A suspect committed robbery against a lone pedestrian at night, using physical restraint and threats before fleeing with valuables. After the arrest, he confessed and compensated the victim before the hearing. The proceedings focused on sentencing considerations due to clear facts, reflecting post-coercion compliance, and a mitigated prosecution recommendation was issued.
